# *Drosophila* Robo3 guides longitudinal axons partially independently of its cytodomain

**DOI:** 10.17912/micropub.biology.001228

**Published:** 2024-06-01

**Authors:** Jessie Agcaoili, Timothy A. Evans

**Affiliations:** 1 Biological Sciences, University of Arkansas at Fayetteville, Fayetteville, Arkansas, United States

## Abstract

*Drosophila* Robo3 is an axon guidance receptor that regulates longitudinal axon tract formation in the embryonic ventral nerve cord. Robo3 is thought to guide longitudinal axons by signaling repulsion in response to Slit. To test this, we modified the *robo3* locus to express a version of the receptor lacking its cytoplasmic domain (Robo3∆C). We find that longitudinal axon guidance is reduced, but not eliminated, in embryos expressing Robo3∆C. Our results show that Robo3's cytodomain is partially dispensable for its axon guidance activity and suggest that it may guide axons via a mechanism other than direct transduction of Slit-dependent signaling.

**Figure 1. Generation and characterization of the robo3 robo3∆C cytoplasmic deletion variant:**
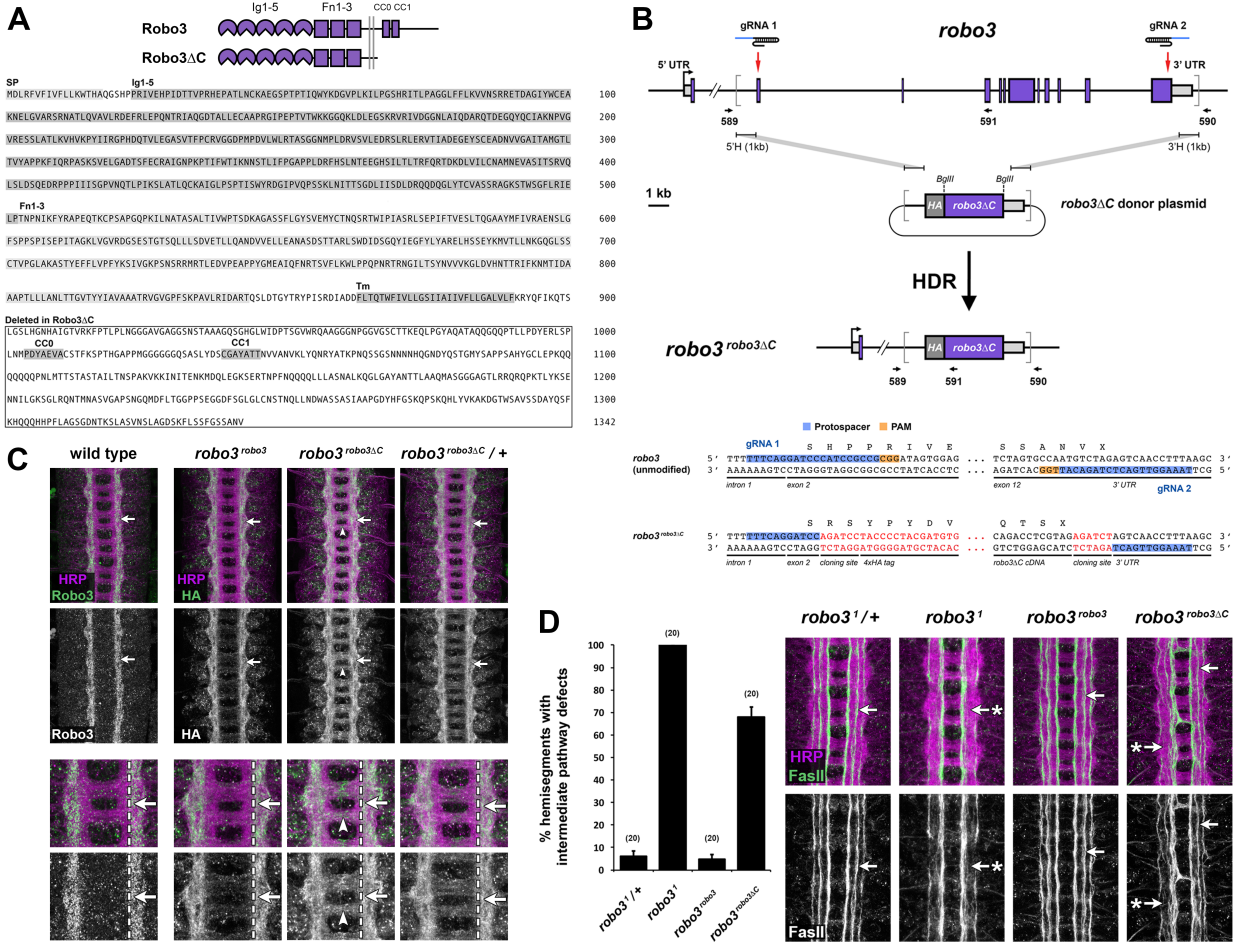
(A) Top, Schematic of the Robo3 protein and the Robo3∆C deletion variant. Robo3 is a single-pass transmembrane protein with an ectodomain containing five immunoglobulin-like (Ig) domains and three fibronectin type III (Fn) repeats. The N-terminal Ig1 domain binds Slit, the common ligand for all three *Drosophila* Robo receptors. The Robo3 cytoplasmic domain includes two conserved cytoplasmic (CC) motifs that are shared among all three *Drosophila* Robos. Bottom, Robo3 protein sequence. The signal peptide (SP), immunoglobulin-like domains 1-5 (Ig1-5), fibronectin type III domains 1-3 (Fn1-3), transmembrane domain (Tm), and conserved cyoplasmic motif (CC0 and CC1) sequences are highlighted. The boxed area shows the sequence that is deleted in the Robo3∆C variant. All but the first 10 amino acids (KRYQFIKQTS) of the cytoplasmic domain are deleted in Robo3∆C. (B) Top, Schematic of the *robo3* gene showing intron/exon structure and location of gRNA target sites, *robo3∆C* homologous donor plasmid, and the modified *robo3 ^robo3∆C^* allele produced via homology directed repair (HDR). Endogenous *robo3* coding exons are shown as purple boxes; 5’ and 3’ untranslated regions (UTR) are shown as light grey boxes. The start of transcription is indicated by the bent arrow. Introns and exons are shown to scale, with the exception of the first intron, from which approximately 13 kb has been omitted. Red arrows indicate the location of upstream (gRNA 1) and downstream (gRNA 2) gRNA target sites. Grey brackets demarcate the region to be replaced by sequences from the donor plasmid. Arrows under schematic indicate the position and orientation of PCR primers. Bottom, Partial DNA sequences of the unmodified *robo3* gene and the modified *robo3 ^robo3∆C^* allele. Black letters indicated endogenous DNA sequence; red letters indicate exogenous sequence. Both DNA strands are illustrated. The gRNA protospacer and PAM sequences are indicated for both gRNAs. The first five base pairs of robo3 exon 2 are unaltered in the *robo3 ^robo3∆C^* alleles, and the *robo3* coding sequence beginning with codon H21 is replaced by the HA-tagged *robo3∆C* cDNA. The endogenous *robo3* transcription start site, ATG start codon, and signal peptide are retained in exon 1. The PAM sequences and portions of both protospacers are deleted in the modified allele, ensuring that the *robo3∆C* donor plasmid and modified *robo3 ^robo3∆C^* allele are not targeted by Cas9. UTR, untranslated regions; 5’H, 5’ homology region; 3’H, 3’ homology region; HA, hemagglutinin epitope tag; gRNA, guide RNA; HDR, homology directed repair; PAM, protospacer adjacent motif. (C) Stage 16-17 *Drosophila* embryonic nerve cords stained with anti-Robo3 (green) or anti-HA (green; detects N-terminal HA tag in modified *robo3* alleles) and anti-HRP (magenta; labels all axons). In wild type embryos, Robo3 protein is detectable on intermediate and lateral longitudinal axons, and excluded from medial longitudinal axons and commissures. HA expression in the full-length *robo3 ^robo3^* gene replacement allele reproduces Robo3’s normal restricted expression pattern. In homozygous *robo3 ^robo3∆C^* embryos, HA-tagged Robo3∆C protein is localized to axons but not restricted to the lateral two-thirds of the longitudinal tracts (arrow). Robo3∆C is also detectable on commissural axon segments in these embryos (arrowhead). Robo3∆C protein distrubution looks normal in heterozygous *robo3 ^robo3∆C^ /+* embryos, although still slightly elevated on commissures. Higher magnification images of the same embryos are shown below, illustrating the expansion of Robo3∆C protein into the medial longitudinal zone (arrow) and commissural axon tracts (arrowhead) in homozygous *robo3 ^robo3∆C^* embryos. For all genotypes shown in (C), Robo3 protein expression was examined in at least three embryos; representative images are shown. (D) Stage 16-17 *Drosophila* embryonic nerve cords stained with anti-FasII (green) and anti-HRP (magenta). All three FasII-positive axon pathways form normally in embryos heterozygous for the *robo3 ^1^* amorphic allele and in homozygous *robo3 ^robo3^* embryos. In homozygous *robo3 ^1^* mutant embryos, axon pathways fail to form in the intermediate zone (arrow with asterisk). In homozygous *robo3 ^robo3∆C^* embryos, intermediate pathways form correctly in around one-third of hemisegments (arrow), while the other two-thirds resemble *robo3* mutants (arrow with asterisk). The extent of defects in *robo3 ^robo3∆C^* embryos is significantly different from both *robo3 ^1^* mutants and *robo3 ^robo3^* embryos (p=1.6e-06 and p=2.0e-12, respectively, by Student's t-test). Some FasII-positive medial axons ectopically cross the midline in *robo3 ^robo3∆C^* embryos, a phenotype that is not observed in *robo3 ^1^* mutants. Bar graph quantifies longitudinal pathway defects in the genotypes shown in (D). Error bars indicate s.e.m. Numbers of embryos scored for each genotype are shown in parentheses.

## Description

During animal development, transmembrane receptor proteins expressed on the surface of axonal growth cones guide axons through the developing nervous system and to their ultimate synaptic targets. These receptor proteins activate intracellular signaling events in response to extracellular cues, promoting local changes to the actin cytoskeleton that steer the growth cone toward or away from secreted attractants or repellants, respectively, or influence cell-cell adhesion to promote or inhibit adherence to membrane-localized substrates (Tessier-Lavigne and Goodman, 1996; Zang et al., 2021) . Roundabout (Robo) family receptor proteins canonically signal midline repulsion in response to their cognate Slit ligands, and this role is conserved across bilaterian animals (Dickson and Gilestro, 2006) . In *Drosophila* , three Robo family proteins are present (Robo1, Robo2, and Robo3) along with a single shared Slit ligand. During embryonic development, Robo1 and Robo2 participate in canonical Slit-dependent midline repulsion in the ventral nerve cord, but Robo3 does not (Rajagopalan et al., 2000a; Simpson et al., 2000a). Instead, Robo3 acts to regulate the medial-lateral position of longitudinal axon tracts, guiding axons away from the medial zone and into the intermediate and lateral zones of the ventral nerve cord neuropile (Rajagopalan et al., 2000b; Simpson et al., 2000b). It has been proposed that Robo3 guides longitudinal axons by signaling repulsion in response to a midline-derived Slit gradient (Rajagopalan et al., 2000b; Simpson et al., 2000b), but more recent evidence indicates that Slit binding is not required for this activity (Carranza et al., 2023) . Other possible mechanisms by which Robo3 could guide longitudional axons include cell-autonomous signaling in response to an unidentified ligand other than Slit, regulating differential adhesion between distinct classes of longitudinal axons by acting as a homophilic or heterophilic adhesion molecule, or acting as a non-signaling member of a heteromeric receptor complex, among others. Some of these candidate mechanisms would likely depend on functional elements within the cytoplasmic domain of Robo3, while others may be cytodomain-independent. Thus, determining whether Robo3's cytodomain is required for longitudinal axon guidance may help distinguish between possible candidate mechanisms.

Robo family receptors are non-catalytic, but a number of evolutionarily conserved short cytoplasmic sequence motifs have been identified in insect, nematode, and vertebrate family members (Bashaw et al., 2000; Daiber et al., 2021; Kidd et al., 1998) . These conserved cytoplasmic (CC) motifs include tyrosine phosphorylation sites and proline-rich sequences that function in receptor regulation and as docking sites for downstream signaling effectors (Bashaw et al., 2000; Fan et al., 2003; Hu et al., 2005; Lundström et al., 2004; Yang and Bashaw, 2006). The two tyrosine-containing CC motifs (CC0 and CC1) are present in all three *Drosophila* Robos, while the two proline-rich motifs (CC2 and CC3) are present in Robo1 but not in Robo2 or Robo3 (Rajagopalan et al., 2000b; Simpson et al., 2000a). While a number of studies have examined the functional importance of cytodomain elements and downstream signaling mechanisms in *Drosophila* Robo1, very little is known about signaling downstream of Robo2 or Robo3 (Chance and Bashaw, 2015; Chaudhari et al., 2021; Coleman et al., 2010) . If Robo3 acts as a cell-autonomous signaling receptor to control longitudinal axon guidance in *Drosophila* , its cytodomain is likely to be important for this activity.

To examine the functional importance of the Robo3 cytodomain, we used CRISPR to engineer the *robo3* locus to express a version of Robo3 lacking its cytodomain (Robo3∆C) ( Figure 1A ). We used two guide RNAs (gRNAs) along with a homologous donor plasmid containing 1 kb upstream and downstream homology regions to replace exons 2-12 of *robo3* with an HA-tagged *robo3∆C* cDNA, creating the *robo3 ^robo3∆C^* allele ( Figure 1B ). We and others have previously used a similar strategy to replace *robo3* with cDNAs encoding full-length wild type *robo3* (Spitzweck et al., 2010) , its *Drosophila* paralogs *robo1* and *robo2* (Spitzweck et al., 2010) , its ortholog from *Tribolium castaneum, TcRobo2/3* (Evans, 2017) , and engineered *robo3* variants in which the Ig1 domain is deleted or otherwise modified (Carranza et al., 2023) . These previous studies demonstrate that replacing exons 2-12 and all of the intervening introns does not impair the expression or function of *robo3* in the embryonic ventral nerve cord, nor does the addition of an N-terminal 4xHA tag interfere with Slit binding or longitudinal axon guidance by Robo3 (Carranza et al., 2023; Spitzweck et al., 2010) .

We first examined the effect of deleting the cytodomain on the expression and axonal localization of the Robo3 protein ( Figure 1C ). In wild type embryos, Robo3 protein is detectable on axons in the ventral nerve cord, and restricted to longitudinal axons in the lateral two-thirds of the neuropile (the intermediate and lateral zones), i.e. Robo3-expressing axons are excluded from the medial zone (Rajagopalan et al., 2000b; Simpson et al., 2000b). Like Robo1 and Robo2, Robo3 protein is excluded from commissural (midline-crossing) axon segments and very little if any Robo3 protein is detectable on commissures. HA-tagged full-length Robo3 protein expressed from the modified *robo3 ^robo3^* allele fully reproduces this expression pattern (Spitzweck et al., 2010) . In embryos homozygous for the *robo3 ^robo3∆C^* allele, Robo3∆C protein is not restricted to the intermediate and lateral zones, and is instead detectable at uniform levels across the entire width of the longitudinal connectives. In addition, Robo3∆C protein is not excluded from commissures, and instead is detectable on midline-crossing and non-crossing axons alike in *robo3 ^robo3∆C^* embryos. These differences in protein expression appear to be due at least in part to misguidance of Robo3-expressing axons, as embryos that are heterozygous for the *robo3 ^robo3∆C^* allele ( *robo3 ^robo3∆C^ /+)* exhibit proper exclusion of Robo3∆C protein from the medial zone and much lower levels of Robo3∆C protein on commissures (though still slightly elevated compared to wild type and *robo3 ^robo3^* embryos). We interpret the presence of Robo3∆C protein on axons in the medial zone as intermediate or lateral Robo3-positive axons inappropriately entering the medial zone, rather than inappropriate expression of Robo3∆C protein on normally Robo3-negative medial axons. Consistent with this interpretation, we show below that FasII-positive intermediate longitudinal axons are also misguided into the medial zone in *robo3 ^robo3∆C^* embryos. We have recently reported a similar expansion of Robo3-positive axons into the medial zone in embryos expressing a version of Robo3 from which the Slit-binding Ig1 domain is deleted *(robo3 ^robo3∆Ig1^ )* (Carranza et al., 2023) .

Next, we examined the axon guidance activity of Robo3∆C by using anti-FasII antibody to label distinct longitudinal axon tracts in the medial, intermediate, and lateral zones of the ventral nerve cord ( Figure 1D ). In wild type embryos or embryos homozygous for the *robo3 ^robo3^* allele, distinct FasII-positive axon pathways form in each of the three zones (Rajagopalan et al., 2000b; Simpson et al., 2000b; Spitzweck et al., 2010) . In *robo3 ^1^* loss of function mutants, intermediate pathways do not form correctly, and axons that normally project within the intermedite zone instead shift towards the midline and merge with pathways in the medial zone. In homozygous *robo3 ^robo3∆C^* embryos, intermediate pathways form correctly in around one-third of ventral nerve cord hemisegments (32.1%), while the other two-thirds (67.9%) resemble *robo3* mutants.

In addition to longitudinal pathway defects, some FasII-positive medial axons ectopically cross the midline in *robo3 ^robo3∆C^* embryos, a phenotype that is not observed in *robo3 ^1^* mutants. We speculate that this may be caused by a dominant-negative-like effect of Robo3∆C expression on Robo1 and/or Robo2, both of which are required for midline repulsion and both of which are expected to be co-expressed with Robo3 in some neurons. A similar effect can be induced through pan-neuronal misexpression of Robo1∆C or Robo2∆C (Evans et al., 2015) . Ectopic midline crossing of some Robo3∆C-expressing neurons would also account for the presence of Robo3∆C protein on commissural axon segments as seen in Figure 1C .

Our results demonstrate that Robo3's ability to guide intermediate longitudinal axons is reduced, but not eliminated, by the loss of its cytoplasmic domain. Thus, any downstream signaling events that are mediated directly by the Robo3 cytodomain are not absolutely required for its role in longitudinal axon guidance. We do not yet know the exact mechanism by which Robo3 guides longitudinal axons, or what other factor(s) may be involved. It is possible, for example, that Robo3 could function in a heteromeric signaling complex with one or more co-receptors, which could compensate for the loss of Robo3 cytodomain-dependent signaling. Our results therefore do not necessarily rule out the possibility that a Robo3-dependent signaling pathway is necessary for guidance of intermediate longitudinal axons, but instead only show that the Robo3 cytodomain itself is not strictly required. Our results further indicate that some sequences within the Robo3 cytodomain do contribute to its longitudinal axon guidance role, since the *robo3 ^robo3∆C^* allele displays reduced function. One possibility is that the reduced activity of *robo3 ^robo3∆C^* is due to loss of the evolutionarily conserved CC0 and CC1 sequence motifs (Rajagopalan et al., 2000b; Simpson et al., 2000a). The CC0 and CC1 sequences are also conserved in *Drosophila* Robo1 and Robo2, which can both substitute for Robo3 to promote intermediate pathway formation (Spitzweck et al., 2010) . Further studies, for example smaller deletions targeting subregions within the cytodomain or engineered point mutations targeting hypothesized phosphorylation sites, will be necessary to precisely define the functional contributions of specific cytodomain sequences in Robo3.

## Methods

***robo3 CRISPR donor and gRNA plasmid cloning***

Construction of the initial *robo3* CRISPR donor and gRNA plasmids was described previously (Evans, 2017) . The *robo3 ^robo3∆C^* cytodomain deletion was generated from the full-length *robo3* donor via PCR using primers 815 and 816 and includes Robo3 amino acids H21-S900 (relative to Genbank/NCBI reference sequence AAF51387) after the N-terminal 4xHA epitope tag.

***Drosophila strains***

The following *Drosophila melanogaster* strains were used: Canton-S (wild type), *robo3 ^1^* (Rajagopalan et al., 2000b), *robo3 ^robo3^* (Spitzweck et al., 2010) , *robo3 ^robo3∆C^* (this study), *w ^1118^ ; sna ^Sco^ /CyO,P{en1}wg ^en11^ (“Sco/CyOwg”), nos-Cas9.P* (Port et al., 2014) *.* All crosses were carried out at 25°C.

***Generation and recovery of CRISPR modified alleles***

The *robo3* gRNA plasmid (Evans, 2017) was co-injected with the *robo3 ^robo3∆C^* homologous donor plasmid into *nos-Cas9.P* embryos (Port et al., 2014) by BestGene Inc (Chino Hills, CA). Injected individuals (G0) were crossed as adults to *Sco/CyOwg* . Founders (G0 flies producing F1 progeny carrying modified *robo3* alleles) were identified by testing two pools of three F1 females per G0 cross by genomic PCR with primers 589 and 591, which produce a 1.6 kb PCR product when the expected modified allele is present (the two primers are 12.1 kb apart in the unmodified *robo3* gene). From each identified founder, 5-10 F1 males were then crossed individually to *Sco/CyOwg* virgin females. After three days, these F1 males were removed from the crosses and tested by PCR to determine if they carried the modified allele. F2 flies from positive F1 crosses were used to generate balanced stocks, and the modified alleles were fully sequenced by amplifying the entire modified region (approx. 5 kb) from genomic DNA using primers 589 and 590, then sequencing the PCR product after cloning via CloneJET PCR cloning kit (Thermo Scientific).

**Immunofluorescence and imaging**

*Drosophila* embryo collection, fixation, and antibody staining were carried out as previously described (Hauptman et al., 2022; Patel, 1994) . The following antibodies were used: Alexa 488-conjugated goat anti-HRP (Jackson ImmunoResearch #123-545-021, 1:500), mouse anti-Fasciclin II (Developmental Studies Hybridoma Bank [DSHB] #1D4, 1:100), mouse anti-βgal (DSHB #40-1a, 1:150), mouse anti-Robo3 cytoplasmic (DSHB #15H2, 1:100), mouse anti-HA (Covance #MMS-101P-500, 1:1000), Cy3-conjugated goat anti-mouse (Jackson #115-165-003, 1:1000). Embryos were genotyped using balancer chromosomes carrying lacZ markers. Ventral nerve cords from embryos of the desired genotype and developmental stage were dissected and mounted in 70% glycerol/PBS. Fluorescent confocal stacks were collected using a Leica SP5 confocal microscope and processed by Fiji/ImageJ (Schindelin et al., 2012) and Adobe Photoshop software.

## Reagents

***Drosophila* strains**

**Table:** 

**Strain**	**Genotype**	**Source**	**Reference**
Canton-S	wild type	BDSC stock #64349	--
*nos-Cas9.P*	y[1] M{w[+mC]=nanos-Cas9.P}ZH-2A w[*]	BDSC stock #54591	(Port et al., 2014)
*Sco/CyOwg*	w ^1118^ ; sna ^Sco^ /CyO, P{en1}wg ^en11^	BDSC stock #1672	--
* robo3 ^1^*	y ^1^ w ^*^ ; robo3 ^1^ /CyO, P{en1}wg ^en11^	BDSC stock #66885	(Rajagopalan et al., 2000b)
* robo3 ^robo3^*	w ^*^ ; TI{TI}robo3 ^robo3^	BDSC stock #97234	(Spitzweck et al., 2010)
* robo3 ^robo3∆C^*	w ^*^ ; robo3 ^robo3∆C^ /CyO, P{en1}wg ^en11^	this study	this study

BDSC: Bloomington *Drosophila* Stock Center.

**Antibodies**

**Table:** 

**Antibody**	**Animal and Clonality**	**Dilution**	**Catalog Number and Source**
Anti-Robo3	Mouse monoclonal	1:100	15H2, DSHB
Anti-HA	Mouse monoclonal	1:1000	MMS101P-500, Covance
Anti-FasII	Mouse monoclonal	1:100	1D4, DSHB
Anti-ß-galactosidase	Mouse monoclonal	1:150	40-1a, DSHB
Anti-Horseradish Peroxidase (Alexa Fluor 488 conjugated)	Goat polyclonal	1:500	123-545-021, Jackson Immunoresearch
Anti-mouse IgG (Cy3 conjugated)	Goat polyclonal	1:1000	115-165-003, Jackson Immunoresearch

DSHB: Developmental Studies Hybridoma Bank.

**Plasmids**

**Table:** 

**Plasmid**	**Catalog Number and Source**
pCFD4-U6:1_U6:3tandemgRNAs	Addgene plasmid #49411 (Port et al., 2014)
*robo3∆C* donor	this study

**Oligonucleotide primers**

**Table:** 

**Primer #**	**Name**	**Sequence (5' to 3')**
589	robo3 CRISPR upstream fwd	TCATGCCTCAAGATCAATTCGATTGATTCC
590	robo3 CRISPR downstream rev	GGGACGGCTAGTTTGCATCTAAATAAGGCC
591	robo3 Ig2 rev	CGCCTGGAGCCGCAGAACACACGGATCGCC
815	robo3dC fwd	GCAGACCTCGTAGAGATCTAGTCAACCTTTAAGC
816	robo3dC rev	TAGATCTCTACGAGGTCTGCTTGATAAATTGGTAGCG
